# The risk of chronic kidney disease in relation to anthropometric measures of obesity: A Swedish cohort study

**DOI:** 10.1186/s12882-021-02531-7

**Published:** 2021-10-05

**Authors:** Ensieh Memarian, Peter M. Nilsson, Isac Zia, Anders Christensson, Gunnar Engström

**Affiliations:** 1 Department of Clinical Sciences in Malmö, Lund University, Internal Medicine – Epidemiology Research Group, Jan Waldenströms gata 15, 5th floor, Malmo, Sweden; 2 Department of Nephrology, Skåne University Hospital, Lund University, S-20502 Malmo, Sweden

**Keywords:** Anthropometric measures, BMI, Chronic kidney disease, Obesity

## Abstract

**Background:**

It has been shown that individuals with obesity have a higher risk for chronic kidney disease (CKD). However, it is unclear which measure of obesity is most useful for prediction of CKD in the general population. The aim of this large prospective study was to explore the association between several anthropometric measures of obesity, i. e., body mass index (BMI), waist circumference (WC), waist circumference to height ratio (WHtR), waist-to-hip ratio (WHR), percentage of body fat (BF%), weight, height and incidence of hospitalizations due to CKD, in a population-based cohort study.

**Methods:**

The ‘Malmö Diet and Cancer Study (MDCS)’ cohort in Sweden was examined during 1991 to 1996. A total of 28,449 subjects underwent measurement of anthropometric measures and blood pressure and filled out a questionnaire. Incidence of in- and outpatient hospital visits for CKD was monitored from the baseline examination over a mean follow-up of 18 years. Cox proportional hazards regression was used to explore the association between anthropometric measures and incidence of CKD, with adjustments for risk factors.

**Results:**

The final study population included 26,723 subjects, 45-73 years old at baseline. Higher values of BMI, WC, WHR, WHtR and weight were associated with an increased risk of developing CKD in both men and women. Only in women, higher values of BF% was associated with higher risk of CKD. Comparing the 4^th^ vs 1^st^ quartile of the obesity measure, the highest hazard ratio (HR) for CKD in men was observed for BMI, HR 1.51 (95% CI: 1.18-1.94) and weight (HR 1.52 (95% CI: 1.19-1.94). For women the highest HR for CKD was observed for BF%, HR 2.01 (95% CI: 1.45-2.78).

**Conclusions:**

In this large prospective study, all anthropometric measures of obesity were associated with a substantially increased incidence of CKD, except for BF% in men. Some measures were slightly more predictive for the risk of CKD than others such as BMI and weight in men and BF% in women. In clinical daily practice use of all anthropometric measures of obesity might be equally useful to assess the risk of developing CKD. This study supports the strong evidence for an association between obesity and CKD.

**Supplementary Information:**

The online version contains supplementary material available at 10.1186/s12882-021-02531-7.

## Background

Worldwide, around 8–16 % of populations are suffering from chronic kidney disease (CKD) [1]. CKD is associated with a significantly higher risk of cardiovascular events, hospitalization and mortality, and the risk increases with the severity of CKD [1–4]. Many factors act synergistically in the development and progression of CKD, most importantly diabetes and hypertension [1]. Obesity, defined as a body mass index (BMI) ≥ 30 kg/m^2^, is associated with substantially increased incidence of type 2 diabetes and hypertension [5]. Notably, individuals with obesity who do not have metabolic abnormalities, also have a higher risk for CKD progression through other mechanisms than hypertension and diabetes [6].

BMI is the most practical and commonly used anthropometric measure for general adiposity [7–9]. However, BMI cannot differentiate between visceral fat and subcutaneous fat. Compared with subcutaneous fat, visceral fat has higher metabolic and inflammatory activity [10, 11]. Other anthropometric indicators that reflect obesity are waist circumference (WC), waist-to-hip ratio (WHR), percentage of body fat (BF%), and waist circumference to height ratio (WHtR). According to a recent meta-analysis, WHtR could be a better predictor for CKD risk relative to other obesity measurements [12]. However, only one study (from Japan) had a prospective design and the authors concluded that high-quality prospective studies are needed to verify the usefulness of WHtR [12]. Another study from China indicated that BF% was a better measure to predict CKD development than other adiposity indices [13].

The *aim* of this large prospective study was to explore the association between several anthropometric measures of obesity, i. e., BMI, WC, WHtR, WHR, BF%, weight, height and incidence of hospitalizations due to CKD, in a population-based cohort study.

## Methods

### Study population

In the present study, we used the ‘Malmö Diet and Cancer Study (MDCS)’ cohort, from the city of Malmö in southern Sweden. Subjects in this study were men born between 1923 and 1945, and women born between 1923 and 1950, living in the city of Malmö, who were invited to participate in the MDCS baseline screening during 1991 to 1996, the participation rate was 41% [14, 15]. A total of 28,449 subjects underwent measurement of anthropometric measures and blood pressure and filled out a questionnaire. The anthropometric measures were obtained only once at the baseline screening during 1991 to 1996.

Subjects with a diagnosis of CKD at baseline were excluded (N=36). Subjects with missing values of anthropometric measurements and lifestyle, socioeconomic and other biological variables were also excluded (n=1688). Additionally, two subjects with mismatched BMI and waist circumference were excluded. The total population included in this study was 26,723 (10,264, 38.4% men, and 16,459, 61.6% women) individuals, aged 45–73 years.

### Baseline examinations

The baseline examinations were performed by qualified nurses at the study center. Height in centimeter (cm) was measured in standing position with a fixed stadiometer. Weight was measured using balance-beam scale, to the nearest 0.1 kg, the subjects were wearing only light clothing and they were not wearing any shoes. BMI (kg/m^2^) of the participants was calculated as weight (kg) divided by the square of the height (m^2^). Participants’ WC (cm) was defined as the circumference between the lowest rib margin and iliac crest, and hip circumference was determined as the largest circumference between waist and thighs. The ratio of WC to hip circumference was considered WHR. The ratio of WC to height was defined as WHtR. These baseline examinations are performed for participants in MDCS cohort and have been mentioned in a study by Borné et al. [16].

Body composition, and BF% was calculated using Bioelectric Impedance Analyzers (BIA), based on the manufacturer instructions (BIA 103, RJL systems, single-frequency analyzer, Detroit, USA) [17]. All the anthropometric measures, i. e. weight, height, BMI, WC, WHR, WHtR and BF%, were categorized into quartiles Q1-Q4 for men and women.

Data on smoking, alcohol consumption, physical activity, educational level, marital status and immigrant status, as well as utilization of lipid-lowering, antihypertensive and diabetes medications, was collected through a questionnaire.

Blood pressure (mmHg) was checked by a nurse using a mercury-column sphygmomanometer after the subjects had rested for 10 minutes in a supine position. Diabetes mellitus was defined as self-reported physician’s diagnosis of diabetes or use of diabetes medications. Physical activity level was identified through 18 questions. The responses were weighted for the intensity of the various activities and added up to a physical activity score [18]. Low leisure-time physical activity was considered as the lowest third of the score.

Subjects’ smoking habits were categorized into two groups, non-smokers (ex-smokers and never-smokers) and smokers (occasional or habitual smokers). High alcohol consumption was considered as an alcohol consumption more than 30 grams per day for women and more than 40 g per day for men. Educational level was divided into three categories: less than 9 years (primary education), 9 - 12 years (some/completed secondary education), and more than 12 years (education at college or university level). Marital status was categorized into two groups; married/cohabitant and single (unmarried/widows/widowers). Country of births was classified as Swedish-born or foreign-born.

A subgroup of MDCS was randomly invited for a cardiovascular study [19]. In this subpopulation 4742 individuals had data available on estimated glomerular filtration rate (eGFR). Serum creatinine and cystatin C was measured in fasting blood samples that was collected at a second visit approximately four months after the first baseline examination. Blood samples were stored at -80 Celsius until analysis in 2012. Creatinine (μmol/L) was measured in plasma and analyzed with the Jaffé method and traceable to the international standardization with isotope dilution mass spectrometry (IDMS). Cystatin C was attained using a particle-enhanced immunonephelometric assay (N Latex Cystatin; Dade Behring, Deerfield, IL, USA). The values for cystatin C were analyzed before the introduction of the world calibrator in 2010 and thus not standardized. The reference value for the method was 0.53–0.95 mg/L. Calculation of eGFR was done according to the previously reported Chronic Kidney Disease Epidemiology Collaboration (CKD-EPI) creatinine–cystatin C equation [20]. A factor of 0.0113 was included to convert creatinine levels measured in μmol/l into mg/dl.

### Incidence of CKD

The subjects included in this study were followed from the baseline examination in 1991-1996 until CKD diagnosis, death, emigration or end of study, December 31^st^, 2016, whichever came first.

Data on incident CKD was retrieved through linkage with the Swedish Cause of Death Register and the Swedish Patient Registers, which covers all inpatient diagnoses in Sweden since 1987 and all hospital outpatient diagnoses since 2001. The registers are managed by the Swedish National Board of Health and Welfare. In addition, the Swedish Kidney Quality Register ‘Svenskt Njurregister’ was used to identify severe CKD, in need of dialysis or transplantation [21].

Diagnoses were coded based on the International Classification of Disease (ICD). The 9th edition (ICD-9) was used during 1987 and 1996 and the 10th edition (ICD-10) from 1997 until present time. CKD was defined as codes 585–586 according to ICD-9, and N18 and N19 according to ICD-10.

A validation study has been performed to assess case validity of the CKD diagnosis from the Swedish patient registers [22]. In 100 randomly selected patients from MDCS, the CKD diagnoses were evaluated by two experienced specialists in nephrology using patient records and laboratory data. A CKD diagnosis requires two measuring points at least 3 months apart to meet the criteria [23] . The diagnoses were divided into four groups based on the degree of reliability: grade 0, incorrect diagnosis; grade 1, low probability of correct diagnosis, or insufficient information (e.g. eGFR 58 ml/min at one time only and no indication of albuminuria); grade 2, reasonable high probability of correct diagnosis (e.g. 2.5 months between creatinine analyzes instead of 3 months); grade 3, correct diagnosis. Grades 0–1 was considered incorrect. Grades 2–3 were considered to be correct. The final result showed 94 percent correct diagnoses, 5 incorrect diagnoses, and 1 case that cannot be classified as correct due to doubtful or insufficient data. Thus, the validation showed that 94% of the patients had a correct diagnosis of CKD.

### Statistical analysis

Cox proportional hazards regression was used to examine the association between anthropometric measures such as BMI, WC, WHR, WHtR, BF%, weight and height (in sex-specific quartiles) and incidence of CKD. The time axis is follow-up time until emigration, incident CKD, death or end of follow-up. Hazard ratios (HR), with 95% confidence interval (CI) was calculated. A p-value < 0.05 was considered statistically significant.

Multivariable Cox proportional hazards models were adjusted as follows: In the basal model, HR were adjusted for age. In the second model, HR were adjusted for age and other biological risk factors such as systolic blood pressure, utilization of antihypertensive or lipid-lowering medications, diabetes mellitus, smoking habits, alcohol consumption and physical activity. In the third model, HR was further adjusted for age, biological risk factors and socioeconomic factors (i. e. marital status, immigration status and education) and eGFR.

In another analysis we categorized BMI and WC according to World Health Organization accepted risk-groups (WHO cut-offs)

BMI (kg/m^2^) was categorized for both sexes as followed:

1. BMI < 18.5 (underweight)
2. BMI 18.5-25 (normal)
3. BMI 25–29.99 (overweight)
4. BMI 30–35 (obese)
5. BMI > 35 (severely obese)

WC was categorized for men and women as followed:

1. WC < 94 for men and WC < 80 for women (normal)
2. WC 94-102 for men and WC 80-88 for women (overweight)
3. WC > 102 for men and WC > 88 for women (obese)

All analyses were performed using SPSS statistics program, version 25 (IBM, Armonk, NY, USA).

The study was conducted in full accordance with the World Medical Association Declaration of Helsinki.

## Results

### Study population

After exclusion of individuals with missing values and those with a prevalent CKD diagnosis at baseline, the final study population consisted of 26,723 subjects (10,264, 38.4% men, and 16,459, 61.6% women). The characteristics of the study population are presented in Table 1, for men and women separately. Except for BF%, mean values of all anthropometric measurements were higher among men compared to women. Moreover, men had higher proportions of hypertension, diabetes, smoking, high alcohol consumption, use of lipid lowering drugs, being married, low physical activity and low education compared to women.

**Table 1:** Characteristics of cohort participants presenting men and women, incident CKD and healthy subjects separately

	Sex-specific characteristics		CKD during follow up	
	Men	Women	CKD	No CKD
Total n (%)	10264 (38.4%)	16459 (61.6%)	1012 (3.3%) Men 590 (5.7%), Women 422 (2.6%)	25711
Incidence of CKD, n (per 1000 p-y)	590 (3.34)	422 (1.41)	1012(2.1)	0
Age at screening (years)	59 ± 7	57± 8	62 ± 7	58 ± 8
Height (cm)	176.5 ± 6.6	163.7 ± 6.1	170.3 ± 9.1	168.5 ± 8.8
Weight (kg)	81.7 ± 12.1	68.0 ± 11.6	79.5 ± 14.4	73.0 ± 13.5
BMI (kg/m2)	26.2 ± 3.5	25.4 ± 4.2	27.4 ± 4.5	25.6 ± 3.9
BF%	20.7 ± 5.0	30.7 ± 5.0	26.4 ± 7.9	26.9 ± 6.9
WC (cm)	93.5 ± 10.0	77.8 ± 10.5	91.3 ± 13.1	83.5 ± 12.7
WHR	0.94 ± 0.06	0.79 ± 0.06	0.90 ± 0.09	0.85 ± 0.09
WHtR	0.53 ± 0.06	0.48 ± 0.07	0.54 ± 0.07	0.50 ± 0.07
Systolic Blood Pressure (mmHg)	144 ± 19	139 ± 20	153 ± 20	141 ± 20
Follow-up period (years)^a^	17.3 ± 5.0	18.2 ± 3.9	17.8 ± 3.6	17.9 ± 4.4
High Alcohol Consumption (> 40/30 gram per day for men/women), n (%)	763 (7.4%)	398 (2.4%)	43 (4.2%)	1118 (4.3%)
Current smoking, n (%)	2952 (28.8%)	4592 (27.9%)	286 (28.3%)	7258 (28.2%)
Current use of antihypertensive medicine, n (%)	1859 (18.1%)	2568 (15.6%)	403 (39.8%)	4024 (15.7%)
Use of Lipid-lowering Drugs, n (%)	348 (3.4%)	297 (1.8%)	62 (6.1%)	583(2.3%)
Diagnosed diabetes, n (%)	531 (5.2%)	506 (3.1%)	140 (13.8%)	897 (3.5%)
Low physical activity, n (lowest quartile) (%)	2603 (25.4%)	4075 (24.8%)	284 (28.1%)	6394 (24.9%)
Married, n (%)	7458 (72.7%)	10000 (60.8%)	692 (68.4%)	16766 (65.2%)
Born in Sweden, n (%) Immigrant to Sweden, n (%)	9033 (88.0%)	14519 (88.2%)	906 (89.5%)	2264 (88.1%)
	1231 (12.0%)	1940 (11.8%)	106 (10.5%)	306(11.9%)
Educational level • Primary school education, n (%) • Secondary school education, n (%) • Higher levels of education, n (%)				
	4646 (45.3%)	6423 (39.0%)	532 (52.6%)	10537 (41.0%)
	2031 (19.8%)	5022 (30.5%)	236 (23.3%)	6817 (26.5%)
	3587 (34.9%)	5014 (30.5%)	244 (24.1%)	8357 (32.5%)

N=number

p-y = person years

CKD= Chronic kidney disease; BMI = body mass index; BF% = bodyfat-percentage; WHR = Waist-Hip Ratio; WC = Waist Circumference; WHtR = Waist-Height Ratio

Values are means ± standard deviation, unless stated otherwise

^a^Follow-up period from screening to emigration or death or last follow-up date (2013-12-31)

The characteristics of the study participants who developed CKD (N=1012) compared to those who did not developed CKD (N=25,711) are also presented in Table 1. Men had higher incidence of CKD (N=590, 5.7%) compared to women (N=422, 2.6%). Except for BF%, the mean values of all anthropometric measurements, were higher in subjects who developed CKD compared to those who did not. In addition, individuals with incident CKD had higher prevalence of hypertension, diabetes and lipid lowering drugs at baseline.

### Risk of CKD in relation to anthropometric measures

Hazard ratios for CKD in relation to anthropometric measures in four quartiles (Q1-Q4) are presented in Tables 2 and 3 for men and women, respectively, and the corresponding Kaplan-Meier plots are presented in Supplementary Figures S2A-S2G and S3A-S3G. Higher values of BMI, WC, WHR, WHtR and weight were associated with an increased risk of developing CKD in both men and women in all three models. Only in women, higher values of BF% was associated with higher risk of CKD in all three models (Q4 vs. Q1 p-value <0.001). Height itself did not show any association with an increased risk of developing CKD in any of the sexes. The highest HRs for CKD in men (4^th^ vs 1^st^ quartile) was observed for BMI, HR 1.51 (95% CI: 1.18-1.94) and weight (HR 1.52 (95% CI: 1.19-1.94). For women the highest HR for CKD in 4^th^ quartile was for BF%, HR 2.01 (95% CI: 1.45-2.78).

**Table 2:** Hazard Ratios for incident CKD in relation to anthropometric measures in men

Quartiles*	Q1	Q2	Q3	Q4	Q4 vs. Q1 p-value**
**BMI** (range, kg/m^2)	< 23.9	23.9 - 26.0	26.0 - 28.1	> 28.1	
CKD^1^	2.2	3.0	3.5	4.7	
HR-1^2^	1	1.23 (0.95-1.60)	1.42 (1.10-1.83)	2.02 (1.58-2.57)	<0.001
HR-2^3^	1	1.13 (0.87-1.47)	1.28 (0.99-1.65)	1.52 (1.18-1.95)	0.001
HR-3^4^	1	1.13 (0.87 - 1.47)	1.28 (0.99-1.66)	1.51 (1.18 -1.94)	0.001
**WC** (range, cm)	< 87	87-93	93-99	>99	
CKD^1^	2.4	2.7	3.6	4.7	
HR-1^2^	1	1.06 (0.82-1.38)	1.39 (1.09-1.78)	1.85 (1.46-2.35)	<0.001
HR-2^3^	1	1.02 (0.79-1.33)	1.24 (0.97-1.59)	1.36 (1.06-1.74)	0.014
HR-3^4^	1	1.02 (0.79 - 1.33)	1.24 (0.97 - 1.59)	1.36 (1.06 - 1.73)	0.016
**WHR** (range)	<0.90	0.90-0.94	0.94-0.98	> 0.98	
CKD^1^	2.6	3.2	3.4	4.2	
HR-1^2^	1	1.26 (0.98 -1.60)	1.36 (1.07-1.73)	1.88 (1.49-2.37)	<0.001
HR-2^3^	1	1.16 (0.91-1.48)	1.10 (0.86-1.40)	1.38 (1.09-1.76)	0.008
HR-3^4^	1	1.15 (0.90-1.47)	1.09 (0.86- 1.39)	1.36 (1.07- 1.74)	0.012
**WHtR** (range)	< 0.49	0.49-0.53	0.53-0.56	> 0.56	
CKD^1^	2.3	2.6	3.6	5.0	
HR-1^2^	1	1.01 (0.78-1.32)	1.38 (1.080-1.77)	1.90 (1.51-2.41)	<0.001
HR-2^3^	1	0.90 (0.69-1.18)	1.19 (0.93-1.53)	1.34(1.05-1.70)	0.020
HR-3^4^	1	0.90 (0.69 -1.17)	1.18 (0.92 - 1.52)	1.32 (1.03 - 1.68)	0.027
**BF%** (range)	< 17.0	17.0-20.0	20.0-24.0	> 24.0	
CKD^1^	3.2	2.7	3.2	4.3	
HR-1^2^	1	0.85 (0.67-1.08)	0.99 (0.78-1.25)	1.37 (1.10-1.70)	0.004
HR-2^3^	1	0.77 (0.61-0.98)	0.85 (0.67-1.07)	1.08 (0.87-1.34)	0.504
HR-3^4^	1	0.77 (0.61 - 0.98)	0.84 (0.67 - 1.07)	1.07 (0.86 - 1.34)	0.537
**Weight** (range, kg)	< 74.0	74.0-81.0	81.0-89.0	> 89.0	
CKD^1^	2.5	3.2	3.5	4.2	
HR-1^2^	1	1.21 (0.94-1.55)	1.38(1.08-1.76)	1.84 (1.45-2.33)	<0.001
HR-2^3^	1	1.17 (0.91-1.51)	1.27(0.99-1.63)	1.50 (1.18-1.91)	0.001
HR-3^4^	1	1.18 (0.92- 1.52)	1.29 (1.00 - 1.65)	1.52 (1.19 - 1.94)	0.001
**Height** (range, cm)	< 172.0	172.0-176.0	176.0-181.0	> 181.0	
CKD^1^	3.7	4.0	2.9	2.9	
HR-1^2^	1	1.15 (0.93-1.42)	0.86 (0.68-1.09)	0.98 (0.78-1.22)	0.851
HR-2^3^	1	1.17 (0.94-1.45)	0.93 (0.73-1.18)	1.06 (0.85-1.33)	0.608
HR-3^4^	1	1.17 (0.94 - 1.46)	0.95 (0.75 -1.20)	1.09 (0.87 - 1.38)	0.450

*Each quartile includes approximately 2560 individuals. ** *p*-value < 0.05 is considered significant

^1^CKD per 1000-person years; ^2^Cox-regression Hazard Ratio (HR) adjusted for age (95 % CI); ^3^HR-2 adjusted for age, use of antihypertensive medication, lipid-lowering medication, systolic blood pressure, smoking, alcohol consumption, low physical activity and diabetes (95 % CI); ^4^HR-3 adjusted for HR-2 plus low education, marital status and immigrant status (95 % CI)

*CKD* Chronic kidney disease, *BMI* body mass index, *WC* waist circumference, *WHR* waist-hip ratio, *WHtR* waist-height ratio, *BF %* body-fat percentage, *CI* confidence interval

**Table 3:** Hazard Ratios for incident CKD in relation to anthropometric measures in women

Quartiles*	Q1	Q2	Q3	Q4	Q4 vs. Q1 p-value**
**BMI** (range, kg/m^2)	< 22.5	22.5-24.7	24.7-27.6	> 27.6	
CKD^1^	0.8	1.0	1.3	2.6	
HR-1^2^	1	1.04 (0.74-1.45)	1.23 (0.89-1.69)	2.45(1.83-3.26)	<0.001
HR-2^3^	1	0.99 (0.71-1.39)	1.05 (0.76-1.45)	1.65 (1.23-2.22)	<0.001
HR-3^4^	1	0.99 (0.71-1.39)	1.05 (0.76-1.45)	1.63 (1.21-2.20)	0.001
**WC** (range, cm)	< 70	70-76	76-83	> 83	
CKD^1^	0.8	0.8	1.2	2.9	
HR-1^2^	1	0.87 (0.61-1.25)	1.21 (0.89-1.66)	2.68 (2.03-3.55)	<0.001
HR-2^3^	1	0.79 (0.55-1.14)	1.06 (0.77-1.45)	1.72 (1.29-2.30)	<0.001
HR-3^4^	1	0.79 (0.55-1.13)	1.06 (0.77-1.45)	1.70 (1.27-2.27)	0.001
**WHR** (range)	< 0.76	0.76-0.79	0.79-0.82	> 0.82	
CKD^1^	1.0	1.0	1.5	2.2	
HR-1^2^	1	1.00 (0.73-1.37)	1.43 (1.07-1.92)	2.13 (1.63-2.79)	<0.001
HR-2^3^	1	0.93 (0.67-1.27)	1.22 (0.91-1.64)	1.42 (1.08-1.88)	0.013
HR-3^4^	1	0.92 (0.67-1.27)	1.21 (0.90-1.61)	1.40 (1.06-1.85)	0.018
**WHtR** (range)	< 0.43	0.43-0.46	0.46-0.51	> 0.51	
CKD^1^	0.7	0.9	1.4	2.8	
HR-1^2^	1	1.09 (0.75-1.56)	1.57 (1.12-2.20)	2.97 (2.18-4.05)	<0.001
HR-2^3^	1	0.98 (0.68-1.42)	1.31 (0.93-1.84)	1.85 (1.34-2.56)	<0.001
HR-3^4^	1	0.98 (0.68-1.41)	1.30 (0.92-1.82)	1.82 (1.32-2.52)	<0.001
**BF%** (range)	< 27.0	27.0-31.0	31.0-34.0	> 34	
CKD^1^	0.7	0.9	1.3	2.8	
HR-1^2^	1	1.21 (0.84-1.75)	1.47 (1.06-2.04)	2.98 (2.18-4.07)	<0.001
HR-2^3^	1	1.18 (0.81-1.70)	1.25 (0.90-1.74)	2.03 (1.48-2.81)	<0.001
HR-3^4^	1	1.18 (0.81-1.70)	1.24 (0.89-1.73)	2.01 (1.45-2.78)	<0.001
**Weight** (range, kg)	< 60.0	60.0-66.0	66.0-74.0	> 74.0	
CKD^1^	1.0	0.9	1.4	2.4	
HR-1^2^	1	0.89 (0.64-1.24)	1.32 (0.98-1.76)	2.31 (1.77-3.01)	<0.001
HR-2^3^	1	0.84 (0.61-1.17)	1.17 (0.87-1.57)	1.65 (1.25-2.16)	<0.001
HR-3^4^	1	0.84 (0.61-1.17)	1.18 (0.88-1.58)	1.64 (1.25-2.15)	<0.001
**Height** (range, cm)	< 160.0	160-164.0	164.0-168.0	> 168.0	
CKD^1^	1.9	1.4	1.4	1.1	
HR-1^2^	1	0.80 (0.62-1.04)	0.90 (0.69-1.16)	0.85 (0.65-1.12)	0.252
HR-2^3^	1	0.84(0.65-1.09)	0.94 (0.72-1.22)	0.94(0.71-1.23)	0.634
HR-3^4^	1	0.85 (0.66-1.11)	0.96 (0.74-1.25)	0.97 (0.73-1.28)	0.807

*Each quartile includes approximately 4110 individuals. **p-value <0.05 is considered significant

^1^CKD per 1000-person years; ^2^Cox-regression Hazard Ratio (HR) adjusted for age (95 % CI); ^3^HR-2 adjusted for age, use of antihypertensive medication, lipid-lowering medication, systolic blood pressure, smoking, alcohol consumption, low physical activity and diabetes (95 % CI); ^4^HR-3 adjusted for HR-2 plus low education, marital status and immigrant status (95 % CI)

*CKD* Chronic kidney disease, *BMI* body mass index, *WC* waist circumference, *WHR* waist-hip ratio, *WHtR* waist-height ratio, *BF %* body-fat percentage, *CI* confidence interval

**Fig. 1:**
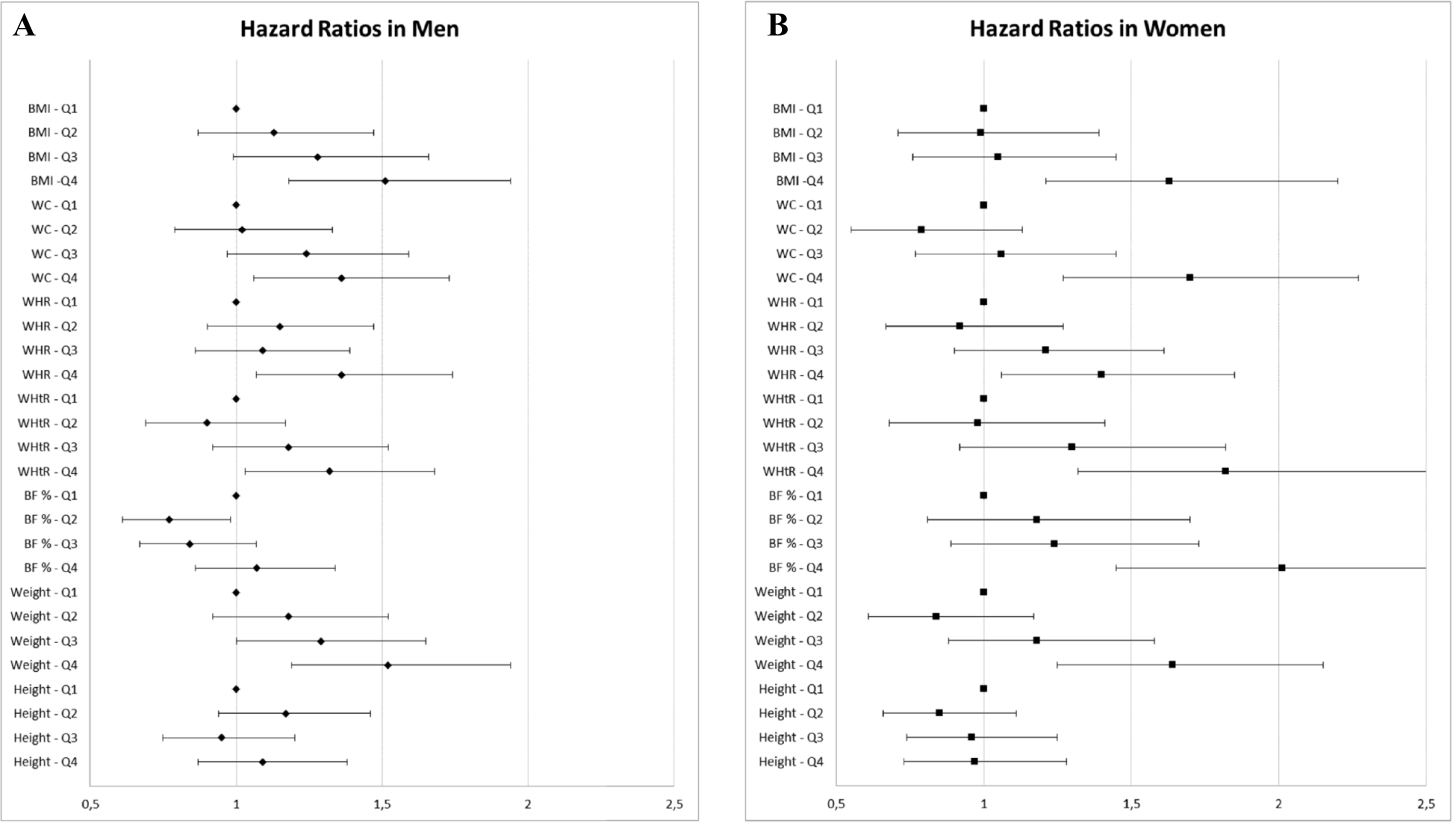
**A** Adjusted HR (95% CI) for different anthropometric measures (in sex-specific quartiles, Q1–Q4) in relation to the risk of developing CKD in men. BMI = body mass index; WC = waist circumference; WHR = waist hip ratio; WHtR = waist height ratio; BF % = body fat percentage; Q = quartile. Graphic representation of Hazard Ratios (HRs) for developing chronic kidney disease (CKD) for each anthropometric measure. HRs are adjusted for age, use of antihypertensive medication, lipid-lowering medication, systolic blood pressure, smoking, low physical activity, diabetes, alcohol consumption, low education, marital status and immigrant status. HRs are represented with a rhomb. Brackets represent 95 % confidence intervals. **B** Adjusted HR (95% CI) for different anthropometric measures (in sex-specific quartiles, Q1–Q4) in relation to the risk of developing CKD in women. BMI = body mass index; WC = waist circumference; WHR = waist hip ratio; WHtR = waist height ratio; BF % = body fat percentage; Q = quartile. Graphic representation of Hazard Ratios (HRs) for developing chronic kidney disease (CKD) for each anthropometric measure. HRs are adjusted for age, use of antihypertensive medication, lipid-lowering medication, systolic blood pressure, smoking, low physical activity, diabetes, alcohol consumption, low education, marital status and immigrant status. HRs are represented with a square. Brackets represent 95 % confidence intervals

Graphic illustrations of hazard ratios for developing CKD for each anthropometric measure in men and women in four quartiles are presented in Fig. 1A and B. HRs are adjusted for age, use of antihypertensive and, lipid-lowering drugs, systolic blood pressure, smoking habits, low physical activity, diabetes, alcohol consumption, low education, marital status and immigrant status. As shown in the Fig, 1A and B, there is a significantly increased risk of developing CKD in the fourth (vs first) quartile for each anthropometric measure, except for height in both men and women.

Supplementary Table S1 and Supplementary Figure S1A and S1B presents the hazard ratios for incidence of CKD in association to commonly used cut-offs for BMI and WC. As shown in this Table, high cut-off values for BMI and WC were associated with higher HRs for CKD.

## Discussion

The results of this large prospective, population-based cohort study showed that higher values of anthropometric measures related to obesity such as BMI, WC, WHR, WHtR and weight in both sexes were major risk factors/markers for incident CKD, independently of several sociodemographic, lifestyle and biological factors. Body height was not associated with risk of CKD in neither men nor women and BF% did not show any association with risk of CKD in men.

Since obesity is an increasing health problem in most countries and a major risk factor for many diseases, it is important to identify and utilize the most informative measures of obesity for preventive purposes. Some observational studies have demonstrated that central obesity, such as WC or WHR, is associated with higher risk of CKD [24, 25], whereas other studies showed that anthropometric measures of general adiposity such as BMI and BF% provide a similar or better risk assessment [13, 26].

Although all obesity measures in our study were associated with higher risk of CKD, overall body weight in men, i. e. BMI and weight, tended to be more predictive for risk of CKD than measures of abdominal obesity, i. e. WC, WHR and WHtR. On the other hand, in women BF%, WHtR and WC were more predictive compared to BMI and weight.

Visceral fat is usually considered to be more metabolically active than subcutaneous fat, and thereby a better predictor of cardiometabolic risk [11]. It is therefore reasonable to believe that individuals with more visceral abdominal obesity could also run a higher risk of developing CKD [27]. A recent study by Miyasato *et al*. suggested that visceral fat was the most sensitive obesity indicator for decline in kidney function [28]. However, a recent study showed that CT measures of obesity, such as visceral abdominal fat and subcutaneous adipose tissue, as well as BMI and WC were all significantly associated with kidney function decline, indicating that anthropometric measures of body fat such as BMI and WC appear to provide as consistent estimates of kidney function decline risk as CT measures [26]. Moreover, although advanced imaging technologies such as CT and MRI might provide a better evaluation of visceral adiposity, using them to screen for CKD risk in a large-scale population or in daily clinical practice is unrealistic due to high costs and high radiation exposure.

The incidence of CKD was higher for men compared to women. This might be explained by the fact that men had higher mean values of all anthropometric measurements (except for BF%) compared to women, as well as higher prevalence of hypertension, diabetes and some other risk factors. Our results also showed that some anthropometric measures of obesity were more predictive for men whereas others were more predictive for women, as mentioned above. A study from China showed that visceral adiposity index and lipid accumulation product index, which is based on a combination of WC and blood triglyceride, had higher predictive ability for identifying CKD in women, but not in men, as compared to traditional indices such as BMI [29]. The mechanisms causing gender-specific differences in CKD are not completely known. Sex hormones influence the fat distribution differently, which consequently might affect the association between obesity and CKD. This is supported by data from a study of male mice, which reported renoprotective effects after administration of estrogen [30]. According to a systematic review and meta-analysis by Wang et al., obesity increases the risk for developing CKD in the general population, however obesity in women was associated with higher risk of CKD compared to men [31]. A possible explanation might be that the differences in exposure to sex hormones in combination with obesity may cause the gender differences. Another explanation is that women in general have a higher percent body fat and more adipose tissue than men with the corresponding BMI [32]. Further studies are needed to clarify these differences.

Although many epidemiologic studies have shown that obesity and CKD are positively correlated, the mechanisms by which changes in adiposity affect CKD risk are not entirely clear. The Kaplan-Meier graphs for risk of CVD in relation to the quartiles of each anthropometric measure illustrated that the differences between quartiles first appear after 10 years of follow-up. Although the Cox models present the risk of CKD at any point in time during follow-up, the length of follow- up is essential for predicting the number of CKD outcomes. It is worth mentioning that earlier stages of CKD might be undetected or only diagnosed in primary health care, whereas only those with more advanced stages of CKD are hospitalized and thus included in this study.

Anthropometric variables are generally reliable measures, which can be measured with high precision and only change slowly over time. However, it is obvious that some individuals could have changed their weight and waist measures during the follow-up period. This would most likely dilute the relationships with incidence of CKD, if anything.

One question is whether bioelectrical impedance has similar validity and reliability. A validation study of the equipment used in the present study concluded that the validity and reliability was acceptable, and that the method could be of value both for clinical use and field studies [33]. However, others have questioned the precision of the bioelectrical impedance method, especially for individuals with body mass indices greater than 34 kg/m^2^ [27]. These individuals often have a relatively high amount of extracellular water and total body water, which might result in an overestimation of fat-free mass and an underestimation of fat mass. Central body fat generally overestimates the percentage of fat-free mass and underestimates the percentage of fat mass in adults with overweight and obesity [34]. These limitations of the bioelectrical impedance method argue in favor of traditional anthropometric measurements for the assessment of obesity and risk of CKD.

### Strengths and limitations

One of the important strengths of this study is the large population-based cohort and a long follow-up period with accumulation of many CKD events. All the subjects have been followed through the Swedish health care system, which has a complete documentation through national population-based registers. Another strengths of this study is that a separate validation study has been performed to assess case validity of the CKD diagnosis from the Swedish patient registers, which showed that 94% of the patients had a correct diagnosis of CKD [22].

One potential study limitation might be that obese individuals, due to co-morbidities, seek medical care more often than non-obese individuals, resulting in more diagnosed cases of CKD in obese individuals. CKD might be undetected for a long time before the individual seeks medical care and cases that did not seek medical care might have been missed. However, this should be largely similar for different measures of obesity.

Another issue to be addressed is whether the study population was representative of the background population since the participation rate of MDCS was approximately 41% [14]. However, a previous health survey from the region, from the city of Malmö, with a 75% participation rate, showed no substantial difference in basic characteristics, such as smoking and obesity, between participants in the MDCS and the health survey [15].

The anthropometric measurements were performed once at the start of the follow up and we had no data about changes in obesity measures during the follow up period. However, anthropometric measures usually change slowly over time. Even though some individuals assumingly changed their weight and waist measures during the follow-up period, this would most likely dilute the relationships with incidence of CKD.

The results were adjusted for systolic blood pressure and anti-hypertensive medication at the baseline examination. It can similarly be assumed that blood pressure and treatment of hypertension often changed during the follow-up. This should also bias the results towards null, if anything. Another limitation is that no information about proteinuria was available. The study design was appropriate to evaluate incidence of hospitalizations due to CKD, but we could not evaluate the progression of impaired kidney function.

Further studies are warranted to investigate whether interventions to treat obesity, such as liraglutide or bariatric surgery, can also reduce risk of CKD.

## Conclusion

In conclusion, in this large prospective study, all anthropometric measures of obesity were associated with a substantially increased incidence of CKD, except for BF% in men. Some measures were slightly more predictive for the risk of CKD than others such as BMI and weight in men and BF% in women. In clinical daily practice use of all anthropometric measures of obesity might be equally useful to assess the risk of developing CKD. This study supports the strong evidence for relation between obesity and CKD risk.

## Supplementary Information

**Additional file 1: Figure S1A**. WHO BMI and WC cut-off specific hazard ratios in men. **Figure S1B**. WHO BMI and WC cut-off specific hazard ratios in women.

**Additional file 2: Figure S2A-S2G**. Survival analyses, men. Quartile specific (Q1-Q4) CKD-free survival rates for men for each anthropometric measure (2A BMI, 2B waist, 2C Waist-Hip Ratio (WHR), 2D Waist-Height Ratio (WHtR), 2E Bodyfat-% (BF%), 2F Weight and 2G Height. The y-axis describes the outcome (1 = 100 %) and the x-axis the follow-up period measured in years.

**Additional file 3: Figures S3A-S3G**. Survival analyses, women. Quartile specific (Q1-Q4) CKD-survival rates for men for each anthropometric measure (2A BMI, 2B waist, 2C Waist-Hip Ratio (WHR), 2D Waist-Height Ratio (WHtR), 2E Bodyfat-% (BF%), 2F Weight and 2G Height. The y-axis describes the outcome (1 = 100 %) and the x-axis the follow-up period measured in years.

**Additional file 4: Table S1**: WHO cut-offs’ specific Hazard Ratios for incidence of chronic kidney disease in men and women.

## Data Availability

The datasets used and analysed during the current study are available after application to the MDCS steering committee on reasonable request. Email: Anders.Dahlin@med.lu.se.

## Abbreviations

BF%: Percentage of body fat
BIA: Bioelectric Impedance Analyzers
BMI: Body mass index
CI: Confidence interval
CKD: Chronic kidney disease
cm: centimeter
CT: Computerized tomography
eGFR: Estimated glomerular filtration rate
HR: Hazard Ratio
ICD: The International Classification of Disease
N: Number
MDCS: Malmö Diet and Cancer Study
WC: waist circumference
WHO: World health organization
WHR: Waist-to-hip ratio
WHtR: Waist circumference to height ratio
