# Supplementary material for: The risk of chronic kidney disease in relation to anthropometric measures of obesity: A Swedish cohort study
Source: BMC Nephrol. 2021 Oct 5;22:330. doi: 10.1186/s12882-021-02531-7 (PMC8491415; doi:10.1186/s12882-021-02531-7)
Supplement: Supplementary file 1 — Additional file 1: Figure S1A. WHO BMI and WC cut-off specific hazard ratios in men. Figure S1B. WHO BMI and WC cut-off specific hazard ratios in women. [file 12882_2021_2531_MOESM1_ESM.docx]

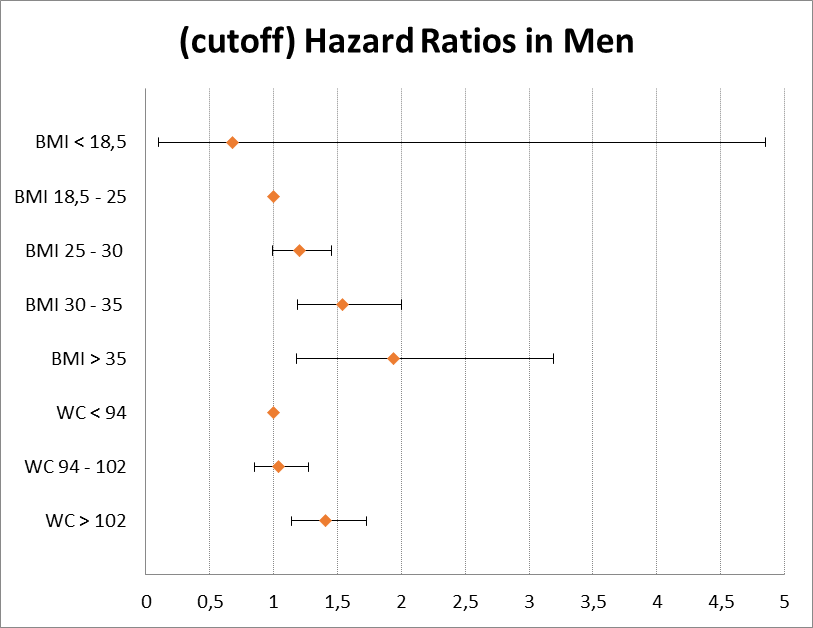


**Supplementary Figure S1A.** WHO BMI and WC cut-off specific hazard ratios in men.

**
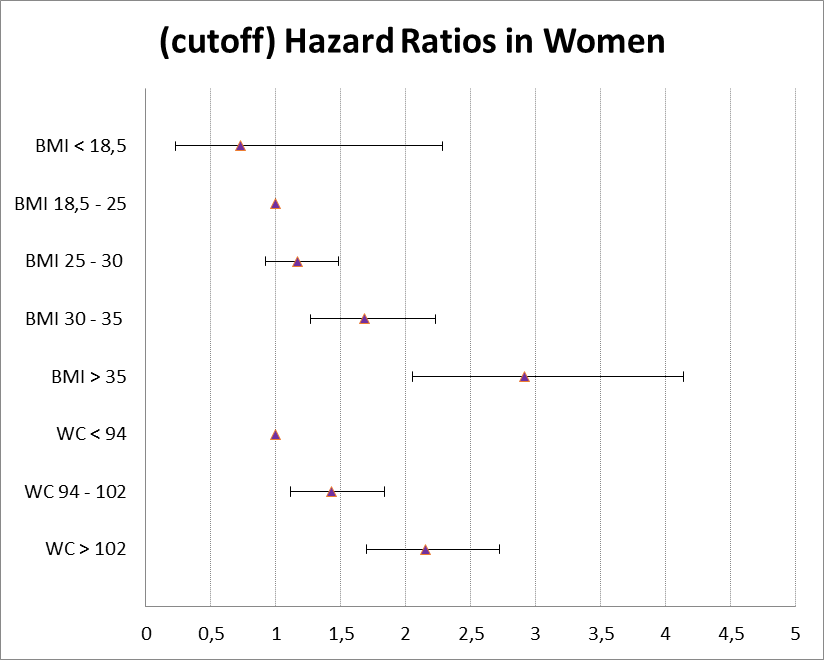
**

**Supplementary Figure S1B.** WHO BMI and WC cut-off specific hazard ratios in women.
