# Supplementary material for: The risk of chronic kidney disease in relation to anthropometric measures of obesity: A Swedish cohort study
Source: BMC Nephrol. 2021 Oct 5;22:330. doi: 10.1186/s12882-021-02531-7 (PMC8491415; doi:10.1186/s12882-021-02531-7)
Supplement: Supplementary file 2 — Additional file 2: Figure S2A-S2G. Survival analyses, men. Quartile specific (Q1-Q4) CKD-free survival rates for men for each anthropometric measure (2A BMI, 2B waist, 2C Waist-Hip Ratio (WHR), 2D Waist-Height Ratio (WHtR), 2E Bodyfat-% (BF%), 2F Weight and 2G Height. The y-axis describes the outcome (1 = 100 %) and the x-axis the follow-up period measured in years. [file 12882_2021_2531_MOESM2_ESM.docx]

**Supplementary Figure S2A-S2G Survival analyses, men**

Quartile specific (Q1-Q4) CKD-free survival rates for men for each anthropometric measure (2A BMI, 2B waist, 2C Waist-Hip Ratio (WHR), 2D Waist-Height Ratio (WHtR), 2E Bodyfat-% (BF%), 2F Weight and 2G Height. The y-axis describes the outcome (1 = 100 %) and the x-axis the follow-up period measured in years.

Survival analyses are not adjusted for any co-variables.

**
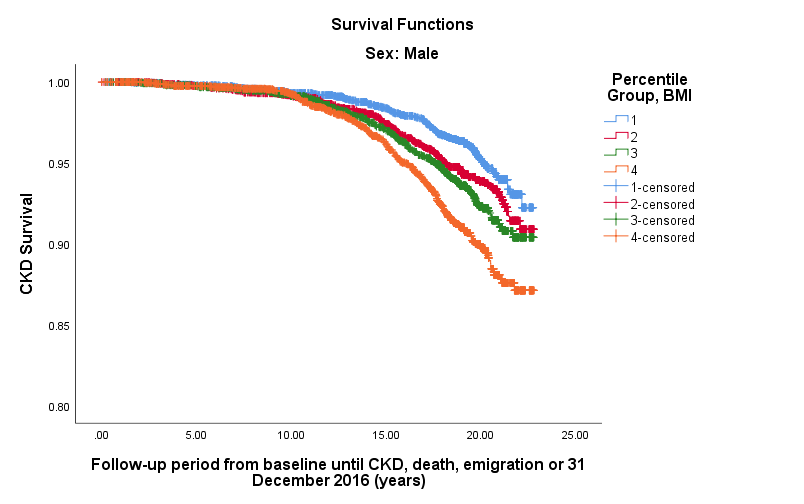
**

**Follow-up (years)**

Sex: male

**CKD – free survival**

**Supplementary Figure 2A**

**
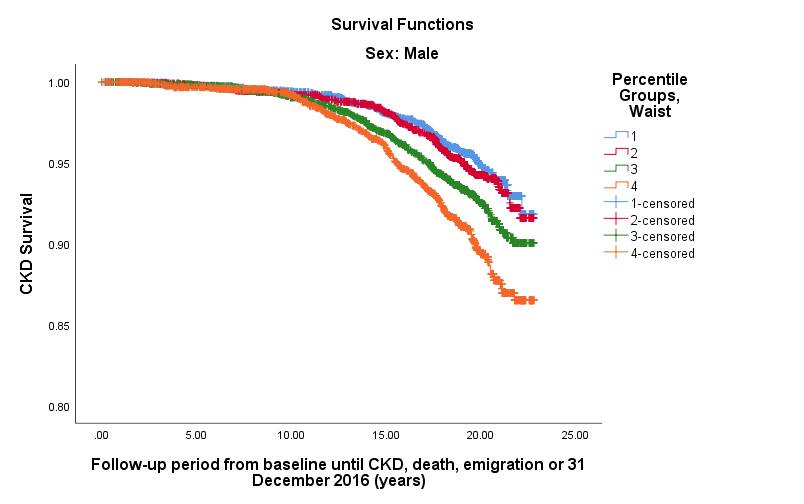
**

**Follow-up (years)**

Sex: male

**CKD – free survival**

**Supplementary Figure 2B**

**
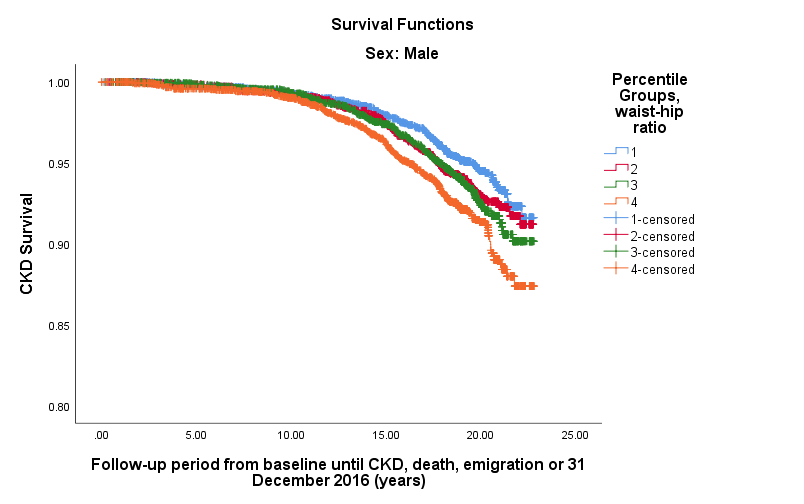
**

**Follow-up (years)**

Sex: male

**CKD – free survival**

**Supplementary Figure 2C**

**
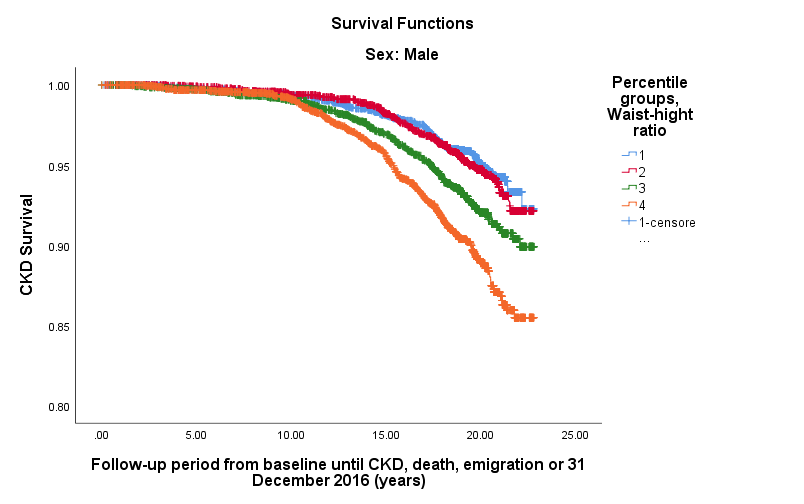
**

**Follow-up (years)**

Sex: male

**CKD – free survival**

**Supplementary Figure 2D**

**
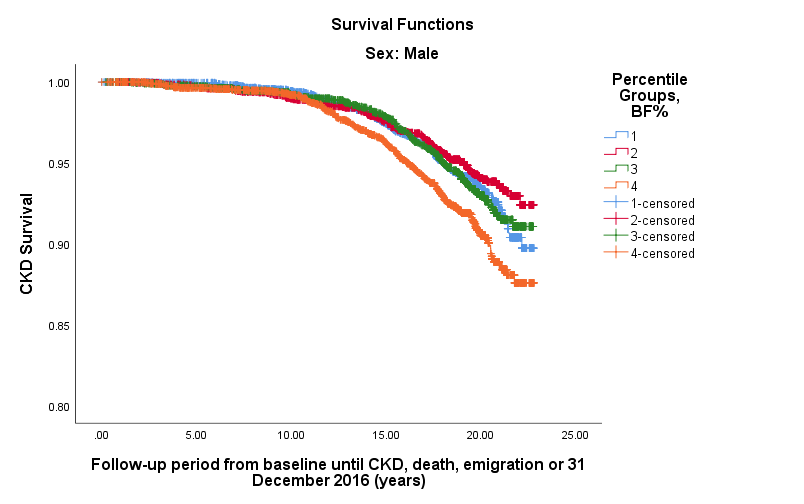
**

**Follow-up (years)**

Sex: male

**CKD – free survival**

**Supplementary Figure 2E**

**
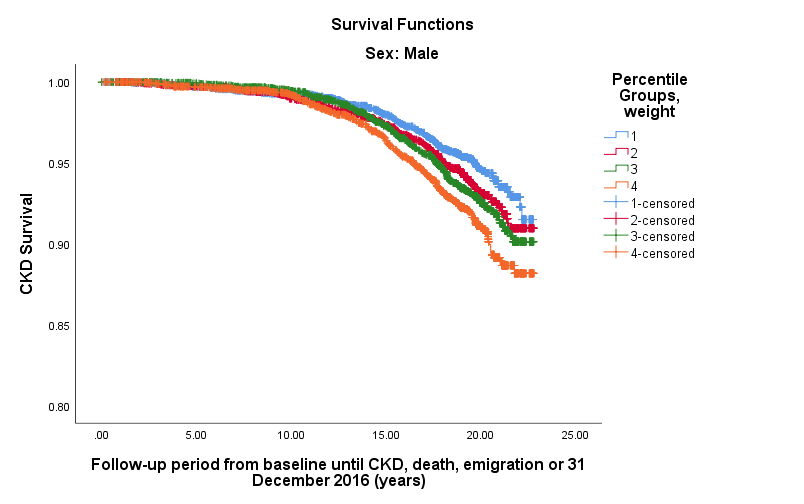
**

**Follow-up (years)**

Sex: male

**CKD – free survival**

**Supplementary Figure 2F**

Sex: male

**
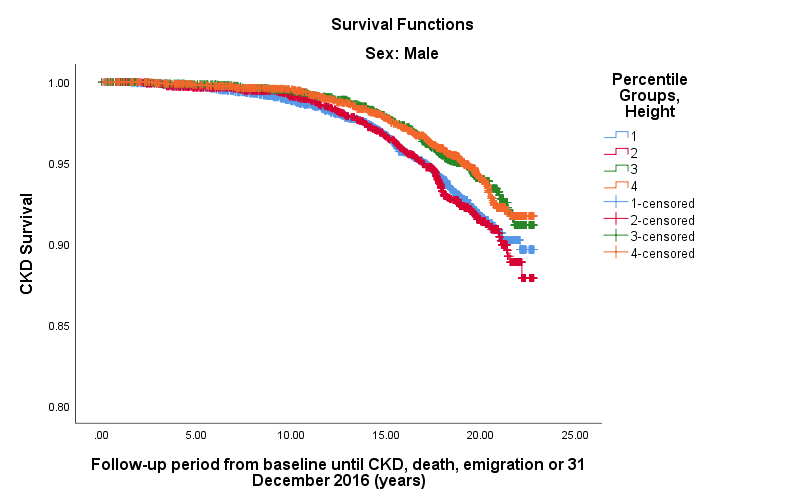
**

**Follow-up (years)**

**CKD – free survival**

**Supplementary Figure 2G**
