# Supplementary material for: The risk of chronic kidney disease in relation to anthropometric measures of obesity: A Swedish cohort study
Source: BMC Nephrol. 2021 Oct 5;22:330. doi: 10.1186/s12882-021-02531-7 (PMC8491415; doi:10.1186/s12882-021-02531-7)
Supplement: Supplementary file 3 — Additional file 3: Figures S3A-S3G. Survival analyses, women. Quartile specific (Q1-Q4) CKD-survival rates for men for each anthropometric measure (2A BMI, 2B waist, 2C Waist-Hip Ratio (WHR), 2D Waist-Height Ratio (WHtR), 2E Bodyfat-% (BF%), 2F Weight and 2G Height. The y-axis describes the outcome (1 = 100 %) and the x-axis the follow-up period measured in years. [file 12882_2021_2531_MOESM3_ESM.docx]

**Supplementary Figures S3A-S3G Survival analyses, women**

Quartile specific (Q1-Q4) CKD-survival rates for men for each anthropometric measure (2A BMI, 2B waist, 2C Waist-Hip Ratio (WHR), 2D Waist-Height Ratio (WHtR), 2E Bodyfat-% (BF%), 2F Weight and 2G Height. The y-axis describes the outcome (1 = 100 %) and the x-axis the follow-up period measured in years.

Survival analyses are not adjusted for any co-variables.

**
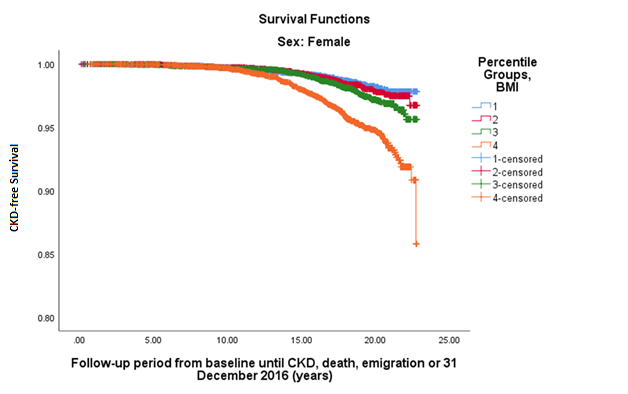
**

Follow-up (years)

Sex: male

**CKD – free survival**

**Supplementary Figure 3A**

**
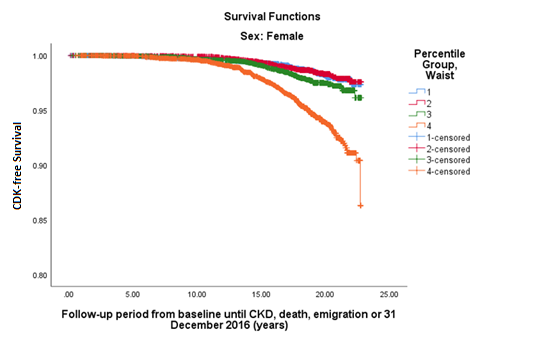
**

**Follow-up (years)**

Sex: male

**CKD – free survival**

**Supplementary Figure 3B**

**
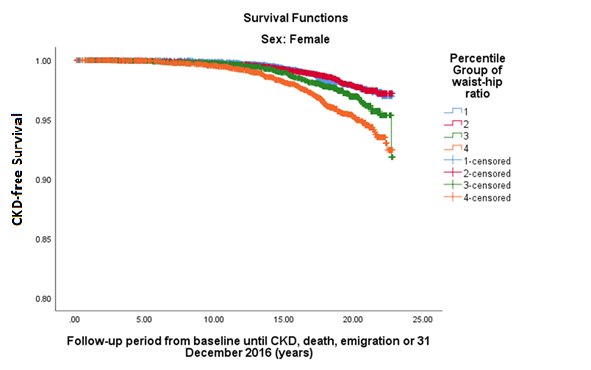
**

**Follow-up (years)**

Sex: male

**CKD – free survival**

**Supplementary Figure 3C**

**
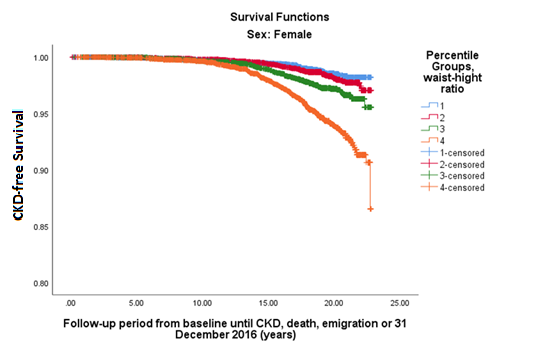
**

**Follow-up (years)**

Sex: male

**CKD – free survival**

**Supplementary Figure 3D**

**
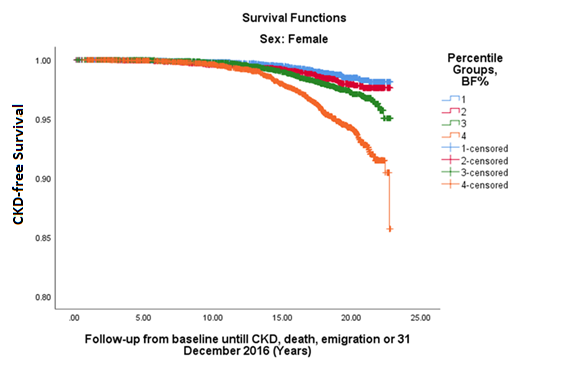
**

**Follow-up (years)**

Sex: male

**CKD – free survival**

**Supplementary Figure 3E**

**
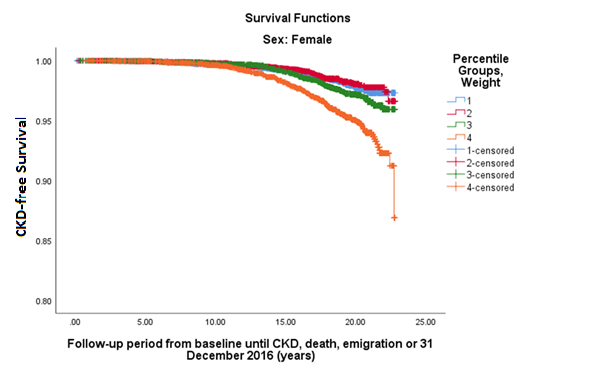
**

**Follow-up (years)**

Sex: male

**CKD – free survival**

**Supplementary Figure 3F**

**
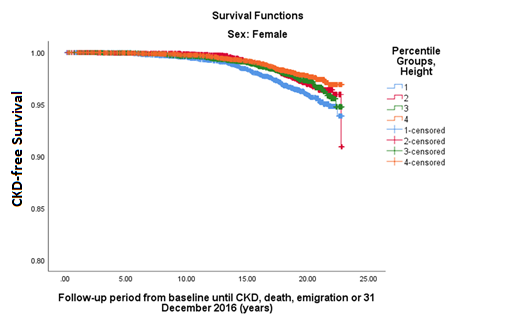
**

**Follow-up (years)**

Sex: male

**CKD – free survival**

**Supplementary Figure 3G**
