# Supplementary material for: The risk of chronic kidney disease in relation to anthropometric measures of obesity: A Swedish cohort study
Source: BMC Nephrol. 2021 Oct 5;22:330. doi: 10.1186/s12882-021-02531-7 (PMC8491415; doi:10.1186/s12882-021-02531-7)
Supplement: Supplementary file 4 — Additional file 4: Table S1: WHO cut-offs’ specific Hazard Ratios for incidence of chronic kidney disease in men and women. [file 12882_2021_2531_MOESM4_ESM.docx]

**Supplementary Table S1:** WHO cut-offs’ specific Hazard Ratios for incidence of chronic kidney disease in men and women.

| **Men** |  | | | | |
| --- | --- | --- | --- | --- | --- |
| BMI cut-offs kg/m^2^ | <18.5 | 18.5-25 | 25-30 | 30-35 | >35 |
| HR^1^ | 0.68 (0.10-4.85) | 1 | 1.20 (0.99-1.45) | 1.54 (1.19-2.00) | 1.94 (1.18-3.19) |
| WC cut-offs | <94 | 94-102 | >102 |  |  |
| HR^1^ | 1 | 1.04 (0.85-1.27) | 1.41(1.14-1.73) |  |  |
| **Women** |  | | | | |
| BMI cut-offs kg/m^2^ | <18.5 | 18.5-25 | 25-30 | 30-35 | >35 |
| HR^1^ | 0.73 (0.23-2.28) | 1 | 1.17 (0.92-1.48) | 1.68 (1.27-2.23) | 2.91 (2.05-4.14) |
| WC cut-offs | <80 | 80-88 | >88 |  |  |
| HR^1^ | 1 | 1.43 (1.11-1.84) | 2.15 (1.70-2.72) |  |  |
| ^1^Hazard ratios (HR) for developing chronic kidney disease, adjusted for age, use of antihypertensive medication, lipid-lowering medication, systolic blood pressure, smoking, low physical activity, diabetes, alcohol consumption, low education, marital status and immigrant status (95% CI).  WHO=world health organisation; BMI=body mass index; HR= hazard ratio; WC=waist circumference | | | | | |
